# Analysis of the Mycotoxin Levels and Expression Pattern of SWN Genes at Different Time Points in the Fungus *Slafractonia leguminicola*

**DOI:** 10.3390/microorganisms12040670

**Published:** 2024-03-27

**Authors:** Sumanjari Das, Dale R. Gardner, Daniel Cook, Rebecca Creamer

**Affiliations:** 1 Department of Biology, New Mexico State University, Las Cruces, NM 88003, USA; das006@gmail.com; 2 USDA Poisonous Plant Research Laboratory, 1150 E 1400 N, Logan, UT 84321, USA; drglogan@gmail.com; 3 Entomology, Plant Pathology, and Weed Science, New Mexico State University, 945 College Ave., Las Cruces, NM 88003, USA

**Keywords:** gene expression, swainsonine, slaframine, *Slafractonia leguminicola*, mycotoxin

## Abstract

The fungal plant pathogen *Slafractonia leguminicola* produces two mycotoxins that affect animals: slaframine, which causes slobbers, and swainsonine, which causes locoism. *Slafractonia leguminicola* contains the swainsonine-associated orthologous gene clusters, “SWN”, which include a multifunctional *swnK* gene (NRPS-PKS hybrid), *swnH1* and *swnH2* (nonheme iron dioxygenase genes), *swnN* and *swnR* (reductase genes), and swnT (transmembrane transporter). In addition to these genes, two paralogs of *swnK*, *swnK1* (paralog1) and *swnk2* (paralog2), are found in *S. leguminicola*. cDNAs from total mRNA were isolated from the *S. leguminicola* mycelia grown in the culture plates as well as from leaves inoculated with the fungal mycelia at different time points, and expression pattern of the SWN genes were analyzed using RT-qPCR. The concentrations of swainsonine and slaframine production from this fungus at different time points were also examined using liquid chromatography–mass spectrometry. The timing of gene expression was similar in cultured fungus and inoculated leaves and agreed with our proposed biosynthetic pathway. Substantially more swainsonine was produced than slaframine during time course studies.

## 1. Introduction

The plant-pathogenic fungus *Slafractonia leguminicola* (formerly named *Rhizoctonia leguminicola*) causes the disease blackpatch of red clover [1,2]. The disease was initially reported from the eastern United States as causing black zonate lesions on leaves and flowers. It is seed transmitted and does not form sexual or asexual spores. The host range includes legumes including several types of clover, alfalfa, cowpea, kudzu, blue lupine, bush bean, lespedeza, and soybean. This Pleosporales plant pathogen produces two mycotoxins, non-living compounds produced as a byproduct of fungal infection, swainsonine, which causes neurological disorders in livestock known as locoism [3], and slaframine, which causes slobbers [4]. In humans, ingestion of plants containing swainsonine can have toxic effects. Locoweed poisoning in livestock is well documented, leading to symptoms; however, direct consumption of locoweed by humans is rare.

Slobbers is primarily a concern for livestock, particularly cattle and horses [5]. There is limited evidence to suggest that slaframine can have direct harmful effects on humans. Typically, humans are not directly exposed to slaframine unless they consume contaminated animal products, such as milk or meat from animals that have ingested slaframine-containing feed. However, the potential effects of slaframine on human health have not been extensively studied. Other than *S. leguminicola*, swainsonine is produced by diverse groups of fungi including endophytes (e.g., *Alternaria oxytropis* from locoweeds, *Alternaria* sp. from *Swainsona canescens*), entomopathogens (e.g., *Metarhizium anisoplae*), and dermophytes (e.g., *Arthoderma otoe*, *Tricophyton benhamiae*) [6,7,8]. Other plant pathogens such as *Alternaria bormuelleri*, which causes a disease of crown vetch, and *Alternaria gansuense*, which causes yellow stunt and root rot of *Astragalus adsurgens*, are morphologically similar to *Alternaria oxytropis* but produce conidia and only trace amounts of swainsonine [9,10,11].

Swainsonine was initially isolated from the plant *Swainsona canscens* from Australia [12] and has since been found associated with other Fabaceae, Convulvaceae, and Malvaceae plants [3,13,14,15,16,17,18]. With the exception of the Malvaceae, species within each have been shown to contain a fungal symbiont that produces the toxin [6,19,20].

Symptoms of locoism include lack of muscular coordination, depression, reproductive problems, impaired vision, difficulty eating and drinking, birth defects, and, in extreme situations, death [21,22,23,24,25]. For horses, the threshold dosage at which clinical signs of locoism appear is considered 0.3 mg of swainsonine per kilogram of animal body weight [26]. Swainsonine is a cytotoxic alkaloid, 1,2,8-trihydroxyoctahydroindolizine, that inhibits α-mannosidase and Golgi mannosidase II, leading to accumulation of sugars in organs, causing neurological damage [27,28]. 

*Slafractonia leguminicola* also produces a second related mycotoxin, slaframine, that causes slobbers syndrome seen in horses and other livestock [4,29,30,31]. Slaframine is specifically produced by *S. leguminicola;* none of the other swainsonine-producing fungi have been reported to produce slaframine [7]. Any slaframine concentrations above 10 ppm may cause clinical signs [5,32]. Slaframine poisoning is not life threatening. Treatment primarily includes removing the horse from the infected hay or pastures.

Animals that feed on slaframine-infested pasture or hay often develop excessive saliva production (slobbering) that is characterized by excessive salivation, lacrimation, feed refusal, bloating, diarrhea, stiffness, weight loss, and sometimes abortion [4,30]. Slaframine toxicoses are particularly pronounced in horses, although toxicoses from both slaframine and swainsonine have often caused comorbidity [33,34,35,36]. Slaframine is an indolizidine alkaloid, 1-acetoxy-6-aminooctahydroindolizine [4,37]. 

Mycotoxin production also varies between *S. leguminicola* and other swainsonine-producing fungi in vivo and in vitro. *Metarhizium* sp. and *S. leguminicola* secrete swainsonine into media (both plates and liquid culture) [38]. Cultures of *S. leguminicola* and *Metarhizium* grow much more rapidly than those of locoweed endophytes, which rarely fill a plate even after a month of growth [39]. 

In addition, *S. leguminicola* produces both swainsonine and slaframine in infected plants [38]. Swainsonine can be found in locoweeds containing *A. oxytropis*, *A. cinerea*, and *A. fulva* in all aerial portions of the plant, with highest levels in the seeds [40]. These endophytes do not cause disease to their plant hosts, nor are they recognized as a pathogen by their hosts [39]. They grow in the pith of stems and do not puncture cell walls. The swainsonine-producing Chaetothyriales fungus associated with *Ipomoea carnea* plants is also not pathogenic and grows endophytically in seeds and epiphytically on leaves [41].

Initial steps in the swainsonine or slaframine biosynthetic pathways were developed from research with *Slafractonia* (recorded as *Rhizoctonia*). These demonstrated steps associated with pipecolic acid and hydroxyindolizidine with the biosynthetic pathways [42,43,44,45,46]. A few protein intermediates were identified in Li et al. [47]. There have been more recent efforts to analyze the beginning steps of the pathway in other fungi [48,49]. In contrast, *Alternaria oxytropis* mutants were screened for important genes using transcriptomic analyses [50]. However, the remainder of the pathway was not reconciled for the fungus until Cook et al. [7]. 

Genome sequence analysis of swainsonine-producing fungi including *Slafractonia leguminicola*, *Metarhizium robertsii*, and *Arthroderma otae* revealed that they share an orthologous gene cluster “SWN” [7], which include seven genes: *swnK*, *swnN*, *swnR*, *swnH1*, *swnH2*, *swnT*, and *swnA*. Neither *Slafractonia* nor any other Pleosporales contained *swnA*, a putative aminotransferase. These genes encode catalytic enzymes involved in SW biosynthesis. *swnK* is a multifunctional gene (NRPS–PKS hybrid) with domains for the initial steps of swainsonine biosynthesis, *swn*N and *swn*R (Rossmann-fold reductases), and *swn*H1 and *swn*H2 (nonheme iron dioxygenases) [7]. The gene *swn*T (a transmembrane transporter) is present in SWN clusters of *S. leguminicola*, *M. robertsii*, and *A. otae* but not in *Alternaria oxytropis* and the Chaetothyriales *Ipomoea carnea* endophyte (ICE) [7,51]. In addition to the SWN gene cluster, *S. leguminicola* possesses two paralogs of *swnK*, *swnK1* and *swnK2*, which may have a role in the biosynthesis of slaframine [6].

A tentative SWN biosynthetic pathway was proposed that predicted the order of the genes for *Metarhizium robertsii* [6] (see Figure 4 in [7]). *SwnK* is proposed to use the initial substrates pipecolic acid and malonyl co-A to produce the primary framework of the swainsonine compound. Then, *swn*R or *swn*N (which were proposed to carry out the same reductase function) reduces the compound to produce 1-hydroxyindolizine. Next, *swn*H1 or *swn*H2 (which were proposed to carry out the same role as dioxygenases) adds oxygens, Lastly, *swn*R or *swn*N reduces the compound to swainsonine.

Other researchers have presented alternative pathways or orders of genes for swainsonine biosynthesis for *Metarhizium* sp. [52] (see Figure 4 in [52]), which suggested swainsonine biosynthesis is multibranched and that *swnA* then *swnR* preceded *swnK* and were followed by *swnN*, then *swnH2*, and lastly *swnH1*. The tentative pathway was developed by knocking out each of the genes in the SWN cluster and determining the consequences on swainsonine and various intermediates. 

Silencing of *swn*T in *S. leguminicola* caused decreased movement of swainsonine and slaframine from mycelia into the culture media [38]. Transformants grew poorly and were unable to infect clover leaves. Deletion of the *swnH2* or *swnH1* gene in *M. robertsii* resulted in the inability of the fungus to produce swainsonine. In contrast, deletion of *swnN*, *swnR*, *swnT*, and *swnA* in *M. robertsii* reduced swainsonine production in varying amounts but did not eliminate it [52]. 

Many orders of fungi have been shown to contain the orthologous swainsonine genes, including Pleosporales (5 fungal species), Onygenales (11 species), Hypocreales (7 species), Xylariales (5 species), and 1 fungal species each from Chaetothyriales, Capnodiales, Microthyriales, Caliciales, Patellariales, Eurotiales, and the Leotiomycetes [51]. Phylogenetic comparisons suggest that in Onygenales and Hypocreales, the SWN cluster was gained once from a common ancestor, while in Pleosporales, it was likely gained several times from one or more common ancestors. Rearrangements and inversions of the SWN cluster appeared to occur within the genera *Metarhizium* (Hypocreales) and *Trichophyton* (Onygenales) as species diverged. Analyses of the intergenic regions showed unique combinations and inversions, including the presence or absence of open reading frames. 

Phylogenetic comparisons showed that the closest match to *S. leguminicola* for *swnK* and *swnR* genes is *Clohesyomyces aquaticus*, another Pleosporales [51]. For *swnN*, *S. leguminicola* grouped with the other Pleosporales. For the *swnH1* and *swnH2* genes, *S. leguminicola* did not cluster closely with any other fungus. Not surprising, the *S. leguminicola swnT* gene did not cluster closely with any other fungus, since no other Pleosporales produced *swnT*. The diversity of SWN genes among the Pleosporales suggests that the SWN genes are likely to have been inherited from different common ancestors. 

The temporal pattern of expression levels of specific SWN genes has been studied in *Metarhizium* [53,54]. These authors reported that *swnT* and *swnK* began high, went down, then went back up. They found that *swnR* began low, went up, and then went back down. This pattern does not match any of the expressions predicted from the synthesis of swainsonine.

While the genes associated with the SWN cluster were identified in *S. leguminicola* [7,51], the order of the genes was not established. This fungus is the only plant pathogen of all the swainsonine producers that has been well characterized. It is also the only fungus that has been shown to produce slaframine. Nothing is known of the two *swnK* paralogs, *swnK1* and *swnK2*, or how their expression might change over time. 

Previous work showed that silencing of *swnT* had a significant effect on *S. leguminicola* growth and pathogenicity and effected secretion of both swainsonine and slaframine [38]. This differed significantly from knockouts of *swnT* in *Metarhizium*, which caused no change in growth of the fungus and no change in secretion. These results together suggest that *S. leguminicola* SWN gene expression may differ from that of *Metarhizium*.

The goal of this study was to investigate the relative expression of the genes in the SWN biosynthesis pathway and also to determine the swainsonine and slaframine concentrations at different growth time points in vivo and in vitro for *S. leguminicola*.

## 2. Materials and Methods

### 2.1. Fungal Strains and Culture Conditions

*Slafractonia leguminicola* (strain: RL-4038, ATCC 26280) was used as the strain for all the studies described herein. Media, potato dextrose agar (PDA) or potato dextrose broth (PDB) was used for routine culture of the fungus at 25 °C. The plates were grown at six different time points (1 d—Day 1, 2 d—Day 2, 3 d—Day 3, 5 d—Day 5, 7 d—Day 7, and 9 d—Day 9). Stock culture of the isolate was maintained on potato dextrose agar (PDA) slants at 4 °C.

### 2.2. Plant Materials

Red clover plants (*Trifolium pratense* L.) were grown from seeds (Source: The Dirty Gardener, Tacoma, WA, USA) and managed routinely in the greenhouse. The plants were about 6 inches tall and were flowering when used for inoculations.

### 2.3. Pathogen Inoculations

The fungi were propagated at room temperature on PDA medium. Detached leaves from approximately 30-day-old red clover plants were inoculated in petri dishes containing a wet filter paper. For inoculation of both culture plates and leaves, a 4 mm plug of 4- to 5-day-old culture was used and maintained in the growth chamber at room temperature for 9 days. 

### 2.4. Total RNA Isolation from the Cultures and the Leaves

Fungal mycelia from the PDA plates or inoculated leaves were collected at different intervals, flash frozen in liquid nitrogen, and stored at –80 °C until used. Total RNA was extracted by TRIzol reagent (ThermoFisher Scientific, # 15596026. Waltham, MA, USA) from both the cultures (100 mg of fungal mycelia) and the leaves (three biological replicates for each time point) following the manufacturer’s instruction. The extracted RNA was run on 1% agarose gel to confirm its integrity. Subsequently, the concentration and purity of the RNA were assessed using a NanoDrop spectrophotometer. All the RNA samples displayed absorbance ratios at A260/280 ranging from 1.9 to 2.0 and at A260/230 around 2.0. Incubating 1 μg of RNA with 1 unit of DNase (DNase I, RNase-free, ThermoFisher Scientific, # EN0521) for 30 min at 37 degrees Celsius, followed by heat denaturation of the enzyme for 5 min at 75 degrees Celsius, effectively eliminated all contaminating DNA. cDNA for subsequent qPCR analysis was prepared from 1 μg of the isolated total RNA using the ProtoScript^®^ II First Strand cDNA Synthesis Kit (New England Biolabs # E6560L, Ipswich, MA, USA) following the manufacturer’s instruction. 

### 2.5. Quantitative Real-Time PCR (RT-qPCR) 

Quantified expression profiling of different transcripts was performed by qPCR on a CFX Connect Real-Time PCR system (Bio-Rad, Hercules, CA, USA) using iTaq Universal SYBR Green Supermix (# 1725121 Bio-Rad) following the manufacturer’s protocol. Primers for amplification of *swnK*, *swnR*, *swnN*, *swnT*, *swnH1*, and *swnH2* are shown in Table 1. The expression of all the genes was normalized to expression for the endogenous control, RDN5.8 [55,56].

PCR conditions were 95 °C for 30 s, followed by 40 cycles of 95 °C for 5 s, 60 °C for 30 s, followed by a melting curve analysis. Then, 50 ng cDNA was used in 20 μL reaction with 400 nM of each primer. RT-qPCR was conducted in triplicate for each sample. RT-qPCR data were analyzed using the 2^−ΔΔCt^ method for relative quantification of gene expression [57]. The differential expression patterns of the *Swn* genes were compared using unpaired Student’s *t* test, with day 1 culture serving as the reference sample for the 2^−ΔΔCt^ analysis.

### 2.6. Determination of Swainsonine and Slaframine Concentrations

Slaframine and swainsonine from the mycelia in the culture (days 1, 2, 5, and 7) and inoculated leaves (days 3, 5, 7, and 9) were measured. The mycelial mass of each culture was dried in the lyophilizer (Labconco, Kansas City, MO, USA) for 2–3 days. Slaframine and swainsonine concentrations were compared using Student’s *t* test. The details of the sample extraction and high-performance liquid chromatography coupled with high-resolution mass spectrometry (HPLC-HRMS) analyses are as follows:

#### 2.6.1. Sample Preparation

Fungal growth on filter paper was cut into small pieces and placed into a tared scintillation vial and weighed. Twenty (20.0) mL of 95% ethanol was added, and the samples were extracted for 16 h with agitation. A portion of the dried agar (100 mg) was placed into a 7 mL screw cap vial, and 5.0 mL of 95% ethanol was added. The samples were extracted for 4 h with stirring. Aliquots of the extracts (0.500 mL from fungal extracts and 0.100 mL from agar extracts) were diluted with an equal volume of deionized water into autosampler vials.

#### 2.6.2. Preparation of Standards

Swainsonine (0.5 mg/mL) and slaframine (0.30 mg/mL slaframine; 1.0 mg/mL as slaframine dipicrate) were diluted (0.02 mL swainsonine solution and 0.033 mL slaframine solution) into 0.95 mL of 50% methanol to give a 10 ppm standard solution. A 0.200 mL aliquot was added to 1.80 mL of 50% methanol to give a 1000 ng/mL solution that was serially diluted to give standards at (1000, 500, 250, 125, 62.5, 31.2, 15.6, 7.8, 3.9, and 1.95 ng/mL). 

#### 2.6.3. HPLC-HRMS Analysis

Standards and sample extracts were analyzed by HPLC-HRMS using the following instrumentation [38]. A Q-Exactive quadrupole/orbitrap high-resolution mass spectrometer (Thermo Scientific) equipped with a heated electrospray ion source (HEIS) and coupled to an Ultimate 3000 HPLC (Thermo Scientific) was used for analyses. Chromatography of samples was performed using a Hypercarb column (100 × 2.1 mm, 5 µm: Thermo Scientific) and a binary mixed solvent system of 20 mM ammonium acetate (A) and methanol (B) flowing at 0.400 mL/min. The gradient mixture was programed as follows: 5% B (0–1 min), 5–70% B (1–5 min), 70% B (5–8 min), 70–5% B (8–10 min), 5% B (10–15 min). Flow from the column was connected directly to an HEIS of the mass spectrometer and calibrated as per the manufacturer’s instructions and with a scan range of 100–800 Da (positive ion), resolution 35,000, micro scans 1, sheath gas flow 35, auxiliary gas flow 10, spray voltage 4 kV, capillary temperature 320 °C, S lens RF field 55, and auxiliary gas temperature 300 °C. Detection and peak areas were obtained from area under the curve of reconstructed ion chromatograms (RICs) of selected ions at *m*/*z* = 174.1122 ± 5 ppm (swainsonine) and *m*/*z* = 199.1437 ± 5 ppm (slaframine). A representative reconstructed ion chromatogram is shown in the Supplemental Files.

## 3. Results

### 3.1. Determination of Swainsonine and Slaframine in the Culture and in the Leaves of S. leguminicola in Different Periods

**Figure 1:**
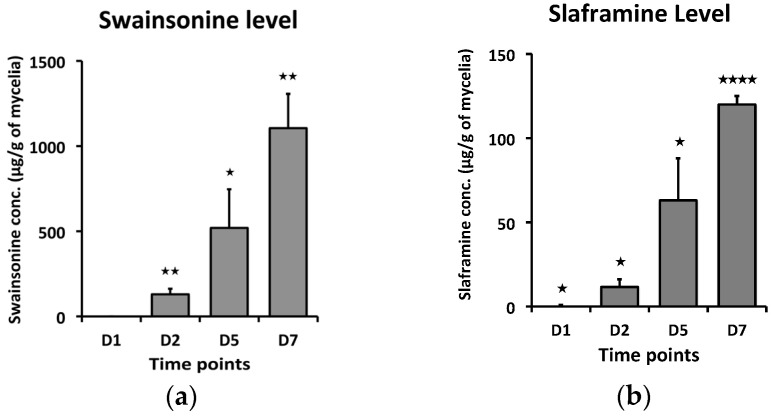
Toxin concentration (µg/g dry mass) at different time points (1D, 2D, 5D, and 7D) for fungus grown on the culture plates. (**a**) Swainsonine concentration with time, (**b**) Slaframine concentration with time. Error bars represent the SEM (n = 3) *: *p* ≤ 0.05, **: *p* < 0.01, ****: *p* < 0.0001 using an unpaired Student’s *t* test.

**Figure 2:**
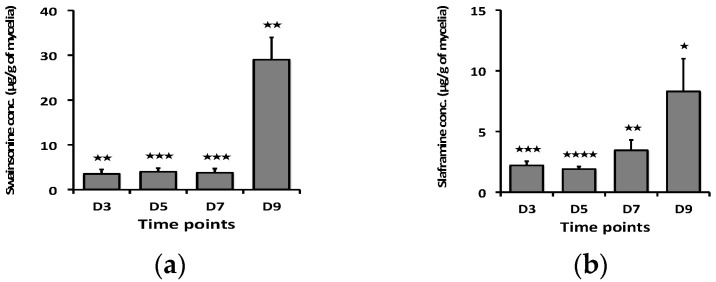
Toxin concentration (µg/g dry mass) at different time points (3D, 5D, 7D & 9D) for inoculated leaves. (**a**) Swainsonine concentration with time, (**b**) Saframine concentration with time. Error bars represent the SEM (n = 3) *: *p* ≤ 0.05, **: *p* < 0.01, ***: *p* < 0.001, ****: *p* < 0.0001 using an unpaired Student’s *t* test.

To detect the toxins, swainsonine and slaframine, content at different time points, a measured amount of *S. leguminicola* culture or leaves infested with the fungus was analyzed by LC–HRMS after sample extraction. In the cultured fungus, there was a statistically significant increase in both swainsonine and slaframine concentrations with time, with 1d showing the least concentration, while at 7d, the concentrations of the toxins were the greatest (Figure 1). In the inoculated leaves, the concentrations of swainsonine and slaframine increased significantly over time, with both toxins reaching a maximum concentration on day 9 (Figure 2).

### 3.2. RT-qPCR Analysis of Key Catalytic Enzyme Genes in the SW Biosynthesis Pathway of S. leguminicola Both In Vitro and In Vivo

**Figure 3:**
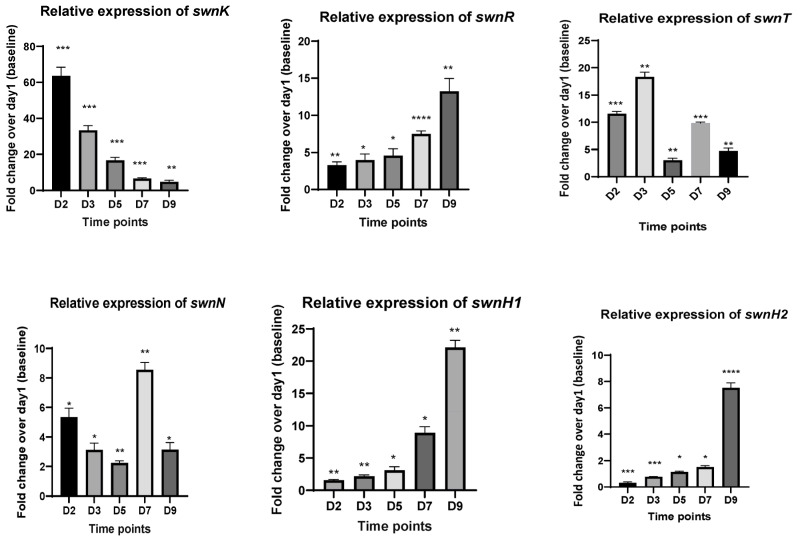
Fold changes in mRNA expression for all genes involved in the swainsonine biosynthetic pathway at different time points. cDNA used for RT-qPCR is synthesized from RNA isolated from the fungal cultures growing on PDA plates at 2d, 3d, 5d, 7d, and 9d. Error bars represent the SEM (n = 3) *: *p* ≤ 0.05, **: *p* < 0.01, ***: *p* < 0.001, ****: *p* < 0.0001 using an unpaired Student’s *t* test.

RT-qPCR was conducted for genes in the SW biosynthesis pathway to determine the relative expression of the genes at 2d, 3d, 5d, 7d, and 9d from the cDNA synthesized from the fungi in the culture plate (PDA) as well as from the detached leaves inoculated with the fungus. In the fungus grown on PDA plates, the expression of *swnK* was upregulated in the early days (1d and 3d) and diminished with time. The expression levels of *swnT* increased at 3d, the expressions of *swnR*, *swnH*_1_, and *swnH*_2_ were downregulated, and the highest gene expression was seen at 9d, while much reduced gene expression was observed in the early days. The expression of the *swnN* gene was significantly upregulated at 2d and 7d (Figure 3). 

**Figure 4:**
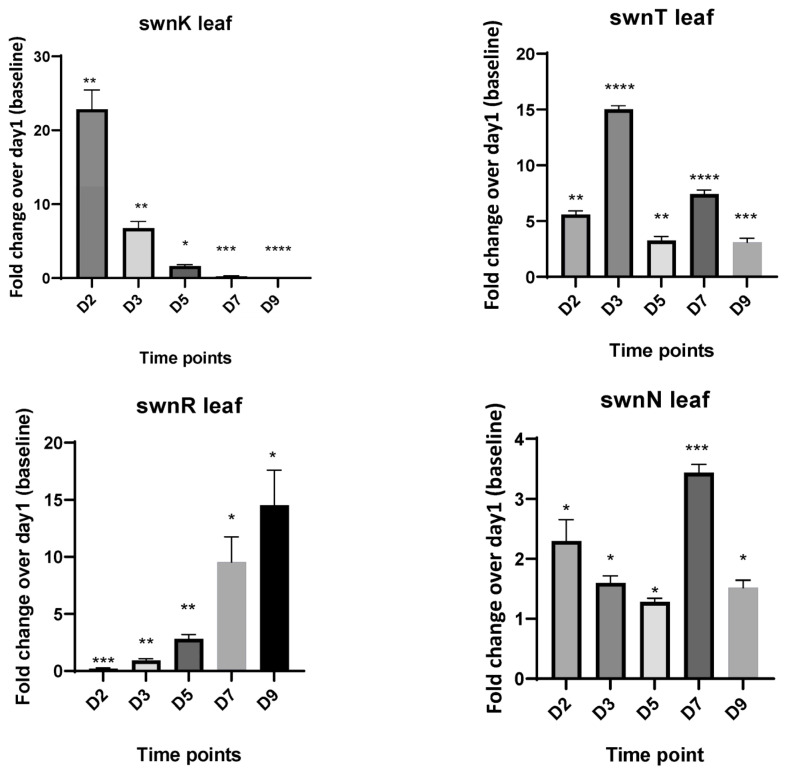

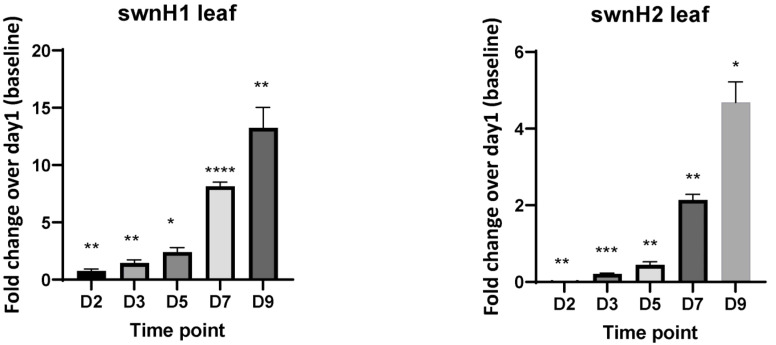
Fold changes in mRNA expression for all genes involved in the swainsonine biosynthetic pathway at different time points. cDNA used for RT-qPCR is synthesized from RNA isolated from the *S. leguminicola* inoculated leaves at 2d, 3d, 5d, 7d, and 9d. Error bars represent the SEM (n = 3) *: *p* ≤ 0.05, **: *p* < 0.01, ***: *p* < 0.001, ****: *p* < 0.0001 using an unpaired Student’s *t* test.

The leaves inoculated with the fungus also showed similar gene expression patterns, though the magnitude of fold change was lower than that seen of the fungi grown on the PDA plates. The *swnK* gene showed highest expression on 2d, and it decreased with time. *swnR*, *swnH1*, and *swnH2* genes showed a gradual increase of gene expression with days, with the maximum expression on 9d. The expression of *swnT* peaked on 3d, and *swnN* showed increased expression on 2d and 7d (Figure 4).

**Figure 5:**
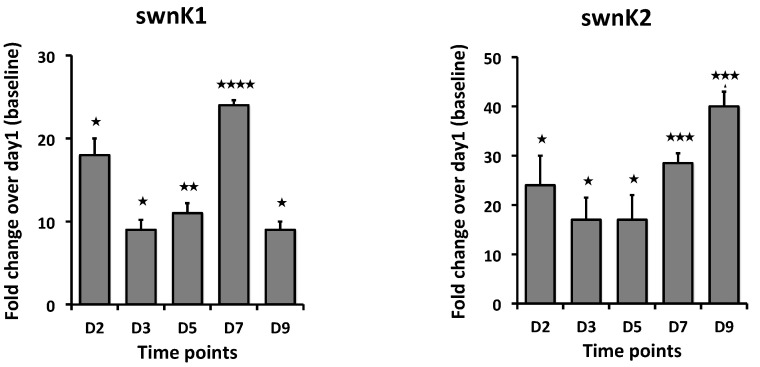
Fold changes in mRNA expression for *swnK* paralogs: *swnK1* and *swnK2*. cDNA used for RT-qPCR is synthesized from RNA isolated from the *S. leguminicola* cultures on PDA plates at 2d, 3d, 5d, 7d, and 9d. Error bars represent the SEM (n = 3) *: *p* ≤ 0.05, **: *p* < 0.01, ***: *p* < 0.001, ****: *p* < 0.0001 using an unpaired Student’s *t* test.

The temporal gene expression pattern was also analyzed for the two paralog genes of *swnK*, *swnK*1 and *swnK*2, which are present exclusively in *S. leguminicola* (5) from the cDNA synthesized from the fungi grown in the culture plates (PDA). The expression level of *swnK1* was higher both at 2d and 7d, with highest expression being on 7d. *swnK2* expression was highest at 9d, while reduced expression was observed on 3d and 5d (Figure 5). 

## 4. Discussion

Our time course of *S. leguminicola* revealed that swainsonine and slaframine concentrations increased with time. The highest concentrations were detected on 7d in the cultured fungus (>1 mg/g dry mass), while in the inoculated leaves, the concentrations spiked on 9d (~3 µg/g dry mass), without much difference in the toxin levels until 7d. The difference in timing might be due to the fact that, in the leaves, the fungus takes time to propagate in the earlier days and then start secreting the secondary metabolites, which are not essential for their pathogenicity. 

In contrast, Huang et al. [53] measured the swainsonine concentration in the fermentation broth of *M. anisopliae* at different timepoints and showed that the content of SW increased at 5d to a maximum of app. 0.3 mg and then decreased. There are many possible reasons for the differences in levels of swainsonine detected and differences in the time courses. These disparities may be because *M. anisopliae* is an entomopathogen within the Hypocreales, while *S. leguminicola* is a plant pathogen within the Pleosporales. The differences between the fungi might also be due to the production of two mycotoxins compared to production of only swainsonine for all other swainsonine-producing fungi. The fungi were grown on different media, and swainsonine was assessed using different methods. In addition, *M. anisopliae* contains an additional gene, *swnA*, not present in any Pleosporales fungus. Comparisons between swainsonine concentrations will be most accurate among different strains of the same fungus grown under the same conditions instead of fungi from different genera, species, and order.

When we compared the swainsonine and the slaframine toxin concentrations of *S. leguminicola*, we found that on 9d of inoculated leaves and 7d for cultured plates, the swainsonine concentration increased to about eight times that of slaframine concentration in the cultured fungus and three times in the inoculated leaves. Producing two toxins may influence the timing and concentration of mycotoxin production by the fungus.

We also studied the temporal gene expression pattern of the key catalytic enzyme genes of the swainsonine biosynthetic pathway in *S. leguminicola*. The SWN gene clusters (*swnK*, *swnN*, *swnH1*, *swnH2*), are involved in the swainsonine biosynthesis pathway, and *swnK* is the key gene [7]. *swnT* acts as a putative transmembrane transporter [38]. We found the pattern of gene expression was similar in the cultured fungus and in the inoculated leaves. The expression levels of *swnK* were upregulated at 2d and decreased after 5d. Levels of *swnT* spiked on 2d and decreased after 3d. The expression of *swnR*, *swnH1*, and *swnH2* were significantly upregulated at 9d. Levels of *swnN* expression gradually decreased after 2d, with a little increase in expression at 7d. 

These results of the RT-qPCR assay agree with the swainsonine biosynthetic pathway proposed in [7]. *swnK* was predicted to be involved upstream in the SW biosynthetic pathway and show higher expression in the initial days, while *swnH1*, *swnH2*, and *swnR* are anticipated to be involved downstream in the process, showing downregulation. The two paralogs of *swnK*, which are present exclusively in *S. leguminicola* and not in any other swainsonine-producing fungi, are predicted to be involved in the slaframine biosynthetic pathway [7]. *swnK1* showed highest expression on 7d, while *swnK2* showed highest expression on 9d. This paralog gene expression pattern is similar to the expression pattern of *swnN*, so it is possible that *swnN* along with either one or both the paralogs are involved in the slaframine biosynthetic pathway, which is yet to be studied.

This temporal gene expression pattern in *S. leguminicola* differs from the pattern of *M. anisopliae* [53,54]. In those studies, the expression levels of *swnT* and *swnK* decreased after 3d and 5d. The relative expression of the *swnR* gene was significantly upregulated at 3d. 

The differences in expression patterns of the genes of the SWN gene cluster in these two different fungi are not surprising. *Slafractonia* is a monotypic species in the order Pleosporales, while *Metarhizium* sp. are in the order Hypocreales. The closest matches for the *swnK*, *swnR*, and *swnN* genes for *Slafractonia* were other Pleosporales, whereas for *M. anisopliae* or *M. robertsii*, the closest matches were other *Metarhizium* sp. Silencing of *swnT* caused a drastic change in the radial growth and morphology of *Slafractonia* and a significant reduction in secretion of swainsonine from mycelia into media. In contrast, knockouts of *swnT* did not affect the growth of *M. robertsii* and reduced swainsonine in both mycelia and media. 

## 5. Conclusions

*Slafractonia leguminicola* is a very unique fungus. It is a member of the Pleosporales that contains the same set of SWN genes, *swnK*, swnR, *swnN*, *swnH1*, and *swnH2*, as the other fungi in the order. It also contains *swnT*, which is unique among the Pleosporales. It produces slaframine in addition to swainsonine. It contains *swnK* and two paralogs, *swnK1* and *swnK2*, that could be involved in the slaframine biosynthetic pathway. *Slafractonia* grows more rapidly that other swainsonine-producing Pleosporales. It is a seed-borne plant-pathogenic fungus that produces mycotoxins in much larger amounts than the other plant-pathogenic Pleosporales that contain the SWN genes. The slaframine and swainsonine play no role in plant pathogenicity but instead cause toxicoses to the animals that feed on the plant hosts of the fungus. 

This study provides information on the timing and relative expression levels of both toxins in plants and in culture. It provides the groundwork for the future study of the swainsonine and slaframine biosynthesis pathways and characterization of the associated catalytic enzyme genes.

## Figures and Tables

**Table 1:** Primers for RT-qPCR of the SWN genes.

Primers	Sequence 5′ → 3′
swnK F	TCGCACAACAAACAGGACAC
swnK R	GACCGCGGAGCTTGCTAAAC
swnN F	AAGAACTCTTGCGCCACCCA
swnN R	AGGCCAACTAAGCGCTCGAT
swnH2 F	AACTTGGCTCACGGAGCTGG
swnH2 R	ACTGCTGCCAAGTCTTTCGT
swnH1 F	AGATTCTCTGCTGGGTCACCA
swnH1 R	TCCCAGGCACATTACGTCCA
swnR F	AGCAGGGTGTTGCCCAGATT
swnR R	CATCTCACGGATGGGCTCGT
swnT F	TCCGGATTGCTTGTCATCTT
swnT R	GATTCACGGCTCAGTGTCCA
RDN5.8 (F)	CTTGGTTCTCGCATCGATGA
RDN5.8 (R)	GGCGCAATGTGCGTTCA

## Data Availability

Data are contained within the article and Supplementary Materials.

## Supplementary Materials

The following supporting information can be downloaded at: https://www.mdpi.com/article/10.3390/microorganisms12040670/s1, Figure S1: Reconstructed Ion Chromatogram of swainsonine and slaframine.
