# Supplementary figures and images for: Analysis of the Mycotoxin Levels and Expression Pattern of SWN Genes at Different Time Points in the Fungus Slafractonia leguminicola
Source: Microorganisms. 2024 Mar 27;12(4):670. doi: 10.3390/microorganisms12040670 (PMC11052177; doi:10.3390/microorganisms12040670)

RT:0.00 - 10.09 SM:5B

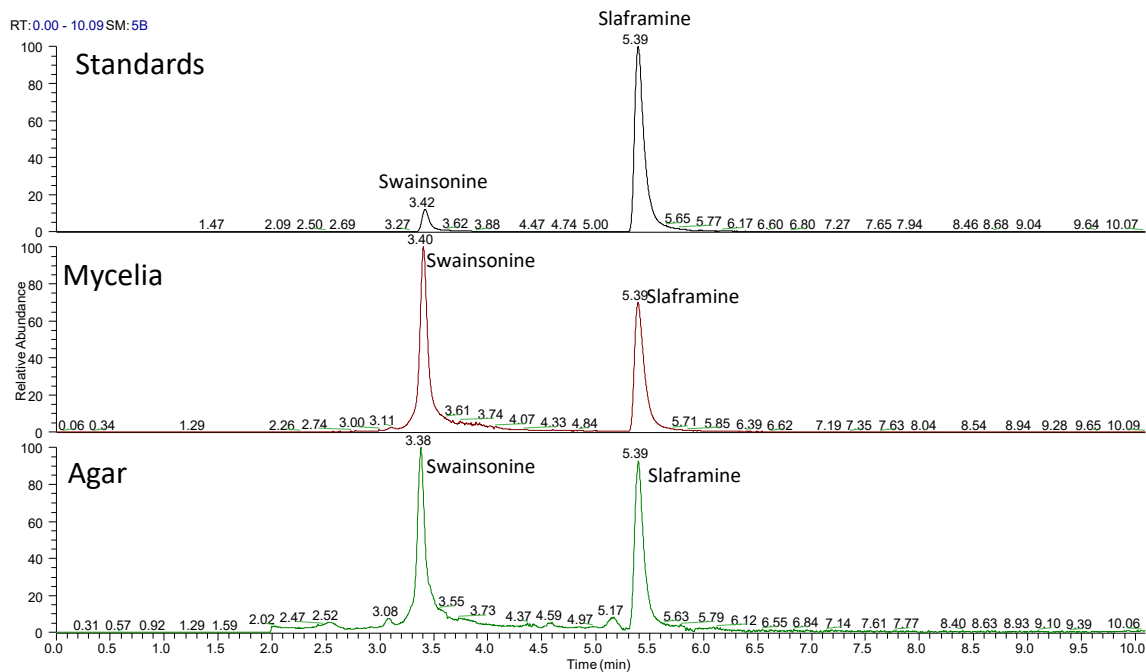

Supplement: Supplementary file 1 [file microorganisms-12-00670-s001.zip › microorganisms-2889297-supplementary.pdf]
